# Assessment of Psychosocial Functioning in a Large Cohort of Patients with Schizophrenia

**DOI:** 10.1007/s11126-020-09773-y

**Published:** 2020-06-09

**Authors:** C. Kossmann, J. Heller, M. Brüne, C. Schulz, M. Heinze, J. Cordes, B. Mühlbauer, E. Rüther, J. Timm, G. Gründer, G. Juckel

**Affiliations:** 1 Department of Psychiatry, Ruhr University Bochum, LWL University Hospital, Alexandrinenstr. 1-3, 44791 Bochum, Germany; 2 Department of Molecular Neuroimaging, Central Institute of Mental Health, Medical Faculty Mannheim, University of Heidelberg, Mannheim, Germany; 3 Brandenburg Medical School, University Clinic for Psychiatry and Psychotherapy, Immanuel Klinik Rüdersdorf, Rüdersdorf, Germany; 4 Department of Psychiatry and Psychotherapy, Medical Faculty, Heinrich-Heine-University, Düsseldorf, Germany; 5 Department of Pharmacology, Klinikum Bremen Mitte, Bremen, Germany; 6 Department of Psychiatry and Psychotherapy, University of Göttingen, Göttingen, Germany; 7 Competence Centre for Clinical Trials – Biometry, University of Bremen, Bremen, Germany

**Keywords:** Schizophrenia, Psychosocial functioning, Recovery, Quality of life

## Abstract

**Background:**

This study addresses the question of whether psychosocial functioning measured by the Personal and Social Performance (PSP) Scale is related to various psychopathological measures in a cohort of patients with schizophrenia.

**Methods:**

The ‘Neuroleptic Strategy Study’ (NeSSy) performed at 14 German hospitals between 2010 and 2013 compared two treatment strategies instead of individual drugs. Secondary end-points were the two PSP scales as well as measures of quality of life (SF-36) and the Positive and Negative Syndrome Scale (PANSS).

**Results:**

149 patients were randomised. There was no difference between the two treatment strategies (first-generation versus second-generation antipsychotics) with regard to the PSP. There were differences in doctors’ assessments regarding psychosocial functioning compared with patients’ own assessments. Furthermore, there were relationships between the PSP and quality of life, level of skills (ICF), and severity of disease (PANSS), level of sexual activities and poor well-being under antipsychotic medication but not with cognitive changes.

**Conclusions:**

The findings on psychosocial functioning of patients with schizophrenia related to severity and skill level could be confirmed. Further findings were the correlation between psychosocial functioning and quality of life, well-being under treatment, and sexuality what emphasizes the substantial importance of a reduced psychosocial functioning.

## Introduction

Psychosocial functioning as the ability to function in family, societal and professional roles takes priority in the life of mentally ill people [1].

An assessment of psychosocial reintegration or psychosocial functioning is useful. Using study data of patients with schizophrenia, it was possible to show that the German translation of the Personal and Social Performance (PSP) Scale is a reliable and valid instrument for recording psychosocial functioning of patients with schizophrenia in acute illness as well as in treatment procedures [2].

The PSP operationalizes psychosocial functioning validly and is as analogically expressive as the Global Assessment of Functioning (GAF) and the scale for measuring social and professional function levels (Skala zur Erfassung des sozialen und beruflichen Funktionsniveaus, SOFAS) scales or the short scale of the international classification of functioning (Internationale Klassifikation der Funktionsfähigkeit, Behinderung und Gesundheit, Mini-ICF-APP). The implementation is efficient. Considering the described disadvantages of the existing scales, PSP is meant to improve upon them [3]. However, in their judgements on psychosocial functioning, patients and doctors only slightly agree.

The validation of the PSP scale in this paper is based on a population of 136 patients with schizophrenia who were registered for the multicentre, randomized, double-blind ‘Neuroleptic Strategy Study’ (NeSSy), carried out with 14 German psychiatric university hospitals and clinics between 2010 and 2013 [4]. Typical (first generation antipsychotics [FGA]) and atypical (second generation antipsychotics [SGA]) antipsychotics were compared. A total of 149 patients with schizophrenia (ICD-10: F20.X) were randomized (FGA: 69; SGA: 80). The data of 136 patients were evaluated. Treatment duration was 24 weeks. In this study (see [4]), the improvement of patient-reported quality of life was significantly higher in patients given SGAs than those given FGAs when treatment selection was individualised. CGI-I values were not different between the two treatment strategies. Psychopathology assessed with the Positive and Negative Syndrome Scale (PANSS) was also not differentially influenced.

The two treatment strategies had disparate effects on socially useful activities as assessed with the PSP with a slight improvement under SGAs and a worsening under FGAs. Disturbing and aggressive behaviour – the one dimension of the PSP that was rated by an observer – was significantly more reduced with FGA treatment than with SGA treatment. Additionally, improvements in aspects of social functioning quantified with the observer-rated part of the PSP were significantly higher with FGA treatment than with SGA treatment, whereas self-rated dimensions of that scale benefited more from SGAs.

The aim in this analysis of the data on psychosocial functioning is to confirm previous findings with a significantly greater number of nationwide cases and to show that the PSP is a good tool for surveying psychosocial functioning.

## Methods

### Patients

Patients ranging from 18 to 65 years old had to present with a diagnosis of schizophrenia (ICD-10: F20.X) to be included in the study. They had to require treatment initiation or change in treatment due to an insufficient response or intolerability to their previous prescribed medication. Exclusion criteria included known hypersensitivity or profound intolerability to one of the study drugs, acute suicidality, treatment on an involuntary legal basis, and the presence of somatic diseases considered by the investigator as significantly interacting with antipsychotic treatment or study procedures. Cannabis abuse was not an exclusion criterion. To avoid selection bias, the presence of tardive dyskinesia did not exclude patients from randomization into an FGA. However, none of the patients included in the study presented with tardive dyskinesia at baseline. Patients had to provide written informed consent to participate in the study.

A total of 136 patients with schizophrenia were registered for the multicentre, randomized, double-blind Neuroleptic Strategy Study (NeSSy) [4] carried out with 14 German psychiatric university hospitals and clinics between April 2010 and May 2013. Typical and atypical antipsychotics were compared on the basis of different therapeutic strategies. A total of 149 patients with schizophrenia (ICD-10: F20.X) were randomized into two groups (FGA: 69; SGA: 80). The data of 136 patients were evaluated.

For this study, 149 patients were recruited, but the full analysis data set for the PSP consisted of only 136 patients because 13 of them did not take part due to drop-out before the administering of drugs (medication) or they did not take the study drugs due to non-compliance. There were 63 patients in the conventional antipsychotic group and 73 patients in the new antipsychotic group. The PSP questionnaires conducted with doctors and patients were completed three times (Visit V0, Visit V4 in week number 6 and Visit V6 in week number 24).

### Sociodemographic Parameters

Out of 136 patients from the full analysis set, 122 filled in the sociodemographic part of the PSP self-rating. Table 1 shows the baseline profiles of 136 patients regarding age, sex, diagnosis, duration, severity of depression (item 6 of PANSS), PANSS total, and baseline profiles of the 122 patients regarding sociodemographic parameters.

**Table 1:** Baseline demographic and clinical characteristics of full-analysis-set (*N* = 136) and sociodemographic parameters completed in PSP Selfrating (*N* = 122)

Baseline demographic and clinical characteristics	FGA (*N* = 63)	SGA (*N* = 73)	Total (N = 136)
Age – year (means ± SD)	35.4 ± 10.6	34.5 ± 10.4	34.9 ± 10.5
Sex – no. (%)			
Male	47 (75)	45 (62)	92 (68)
Female	16 (25)	28 (38)	44 (32)
Diagnosis – no. (%)			
F20	–	2 (3)	2 (2)
F20.0	62 (98)	66 (90)	128 (94)
F20.1	–	1 (1)	1 (1)
F20.3	–	2 (3)	2 (2)
F20.8	1 (2)	1 (1)	2 (2)
Missing	–	1 (1)	1 (1)
Duration of disease – year (means ± SD)^a^	7.1 ± 7.4	4.9 ± 5.9	5.9 ± 6.7
Depression – no. (%)^b^	22 (35)	26 (36)	48 (35)
(Medium – heavy)			
PANSS total score	83.6 ± 16.9	81.4 ± 22.8	82.4 ± 20.2
Sociodemographic parameters	(*N* = 56)	(*N* = 66)	(N = 122)
Occupation – no. (%)			
Fulltime employed	10 (18)	6 (9)	16 (13)
Part-time employed	3 (5)	–	3 (2)
Side job	–	2 (3)	2 (2)
House wife/husband	2 (4)	2 (3)	4 (3)
In education	4 (7)	6 (9)	10 (8)
Student	5 (9)	3 (5)	8 (7)
Unemployed	20 (36)	33 (50)	53 (43)
On pension	8 (14)	11 (17)	19 (16)
Missing	4 (7)	3 (5)	7 (6)
Marital status – no. (%)			
Unmarried, single	37 (66)	38 (58)	75 (61)
Unmarried, Partnership	6 (11)	13 (20)	19 (16)
Married	5 (9)	6 (9)	11 (9)
Divorced	8 (14)	8 (12)	16 (13)
Housing situation – no. (%)			
Living alone	25 (45)	25 (38)	50 (41)
Living with partner/family	10 (18)	14 (21)	24 (20)
Living with parents	10 (18)	15 (23)	25 (20)
Living in shared apartment	7 (13)	7 (11)	14 (11)
Unsettled	1 (2)	1 (2)	2 (2)
Residential home	3 (5)	3 (5)	6 (5)
Missing	–	1 (2)	1 (1)
Assisted living – no. (%)			
Yes	12 (21)	13 (20)	25 (20)
No	39 (70)	48 (73)	87 (71)
Missing	5 (9)	5 (8)	10 (8)

^a^There are two missing values for conventional drugs and three for the newer drugs

^b^There are three missing values for conventional drugs and three for newer drugs

### Study Design

This multicentre, double-blind, double-dummy, randomised study was performed at 14 psychiatric university and state hospitals in Germany. The study was approved by the respective ethics committees and was conducted in accordance with the provisions of the Declaration of Helsinki, the International Conference on Harmonization (ICH), Good Clinical Practice (GCP) guidelines, and current regulatory requirements. Project management, monitoring, data management, and statistical analysis were conducted and supervised by the Competence Centre for Clinical Trials of the University of Bremen.

### Medication

Patients were randomised in a double-blind fashion (random table, block length 30) into two intervention groups, one of which pursued the strategy to apply an FGA and the other the strategy of treatment with an SGA. Two FGAs (haloperidol [3–6 mg] or flupentixol [6–12 mg]) and three SGAs (aripiprazole [10–20 mg], olanzapine [10–20 mg], or quetiapine [400–800 mg]) were selected. Non-psychotropic drugs for the treatment of other medical conditions were allowed throughout the study. All treatments were administered over the duration of 24 weeks until failure of therapeutic response or until the occurrence of major or intolerable adverse events. After cessation of treatment within the study, patients were offered follow-up services for another 24 weeks.

### The PSP and Other Psychometric Scales

Morosini and staff [5] developed the PSP. This assessment is based on four dimensions: socially useful activities, personal and social relationships, self-care, and disturbing or aggressive behaviour.

Compared with the GAF and SOFAS that have been traditionally applied, the PSP implies several advantages. On the one hand, psychopathological symptoms are not mixed with psychosocial aspects on the PSP so that there is a more exact, specific operationalizing of vocational, social, and personal functioning. On the other hand, the four subscales that comprise the total score generated only in the second step are collected beyond a global value. With these four subscales, the PSP creates a higher specific validity than the traditional GAF and SOFAS scales. Furthermore, the scale’s ability to generate quick results must be positively emphasized [5]. In preliminary studies with acute and chronic schizophrenic patients, the scale showed high values of reliability and validity [2, 3]. Thus, the PSP is a third-party assessment tool.

Patient difficulties were rated in each individual section as absent, slight, obvious, distinctive, severe, or extremely severe. After the patient was assessed in terms of the four dimensions, individual values were summarized into a total score; this was done on a scale of 1–100 defined in ranges of 10, where a range must be chosen with individual scores. However, in daily clinical practice the comparison between individual dimensions and therefore different functional areas is of greater practical relevance than the consideration of the total score, since by repeated assessment of the four dimensions in longitudinal section, variations in courses of therapy (short, medium, long) can be detected objectively by repeated assessment of the four dimensions. Additionally, the handling of the PSP is quite easy; only a short training is required.

Table 2 shows the baseline values and pre-post difference for PSP and PSP self-ratings together with the test results. To evaluate secondary target criteria, the difference between V6 and V0 was analysed by default. If the value for visit V0 was missing, the value of V4 was ‘rewritten forward’ First Observation Carried Backwards (FOCB), which means the difference between V6 and V0 was replaced by the difference between V6 and V4. In the case of missing values for V6, the Last-Observation-Carried-Forward (LOCF) method was used, meaning that V6 to V0 was replaced by V4 to V0. If only one single value was available, there was no difference calculation. In some cases, the change from V0 to V4 (short-term development) was interesting; therefore, the difference between V4 and V0 was additionally calculated.

**Table 2:** Baseline and pre-post differenceV6-V0 of PSP and PSP self rating in the FAS

Scores/strategy	Baseline		Pre-post difference V6-V0		*p* value
	N	(Mean ± SD)	N	(Mean ± SD)	
PSP					
Total score					0.8470
FGA	58	51.50 (14.94)	35	5.69 (13.72)	
SGA	66	53.39 (17.02)	37	7.51 (14.68)	
Subscales					
Socially useful activities					0.8931
FGA	59	3.95 (1.07)	37	−0.35 (1.09)	
SGA	68	3.82 (1.23)	42	−0.52 (1.15)	
Personal and social relationships					0.9290
FGA	59	3.32 (1.04)	37	−0.41 (0.90)	
SGA	68	3.25 (1.12)	42	−0.43 (0.94)	
Self-care					0.6843
FGA	59	2.03 (0.93)	37	−0.22 (0.67)	
SGA	68	2.06 (1.09)	42	−0.33 (1.03)	
Disturbing and aggressive behavior^a^					0.1708
FGA	59	1.68 (0.84)	37	−0.32 (0.85)	
SGA	68	1.85 (1.36)	42	−0.14 (0.78)	
PSP Self Rating					
Total score					0.2420
FGA	33	63.58 (23.38)	18	−1.83 (23.89)	
SGA	49	57.12 (20.51)	22	12.32 (25.27)	
Subscales					
Socially useful activities					0.0055
FGA	55	3.26 (1.10)	30	0.39 (0.77)	
SGA	65	3.40 (1.29)	40	−0.17 (1.12)	
Personal and social relationships					0.4844
FGA	55	3.44 (1.17)	30	−0.05 (1.27)	
SGA	65	3.32 (1.29)	39	−0.46 (1.13)	
Self-care					0.7345
FGA	55	2.30 (1.19)	30	0.11 (0.68)	
SGA	65	2.04 (1.07)	39	−0.01 (0.86)	
Disturbing and aggressive behavior					0.2982
FGA	55	2.79 (1.00)	30	−0.11 (0.98)	
SGA	65	2.96 (1.00)	39	−0.41 (0.77)	

^a^Pre-post difference V4-V0 of subscale disturbing and aggressive behavior (*p* = 0.0359)

In addition, the following scales were used: (a) quality of life assessed with the sum of the physical and mental component summary of the Short Form 36 Health Survey (SF-36)15 and the Clinical Global Impression of Severity (CGI-S), or the Clinical Global Impression of Improvement (CGI-I) for sexual dysfunction [6]; (b) Subjective Well-Being Under Neuroleptic Treatment Scale–Short Form (SWN-K); (c) Positive and Negative Syndrome Scale (PANSS) [4]; (d) Mini-ICF-APP (developed by M. Linden); and (e) Mini-ICF-Rating for mental disorders – a short, third-party assessment tool consisting of 12 items that enabled us to operationalize and quantify incapacitating symptoms. The patient’s ability for example to perform daily living or daily structure was assessed with a 5-step Likert-type scale from 0 (*no impairment*) to 4 (*full impairment*) [7].

The assessment of item 9 – ability to participate in family relations – was rated on a 5-step scale for each of the four groups of persons. To obtain a value for item 9, the mean value of all existing values was generated. Likewise, item 10 – ability to perform non-vocational activities – was divided into household chores and recreational activities. Here too, the mean values of both sub-areas were generated before the calculation of the global value. A summarizing assessment of incapacities was confured by generating a mean value over all 12 dimensions according to an overall value [8]. The assessment of cognitive scores is done by the Trail Making Test (A and B) (TMT), the „Regensburg Word Fluency Test’ (RWT), the „Letter-Number-Test’ (BZT), the „Verbal Learn and Memory Test’ (VLMT) and the Multiple Choice Vocabulary Test (MWT-B).

All parameters were recorded at baseline and after 6 (visit 4) and 24 weeks (visit 6). In addition, the primary endpoints and safety parameters were assessed after 2, 4, and 12 weeks (visits 2, 3, and 5).

### Statistical Analysis

Statistical analysis was performed on the full analysis set (FAS) consisting of all randomised patients who received at least one dose of the study drug. The primary efficacy analysis was dedicated to AUC values of SF36 and CGI with a logarithmic time scale from day 1 to week 24, integrating the effect curve with regard to its typical form of early effects flattening out for later visits. Missing values were imputed by LOCF and linear interpolation, respectively. LOCF is more conservative but offers the advantage of implicitly taking into account the time points of dropouts. Secondary tests were always only exploratory. All scores were summarized first in concordance with the original guide, publication, or manual. Correlations were computed by Pearson, respectively, and the Spearman approach, depending on scale properties. The analysis was performed with SAS® version 9.4.

## Results

### Baseline

Table 2 shows that on average, doctors assessed patients as worse regarding total score and activities than patients’ self-assessment. Within the areas of social relationships, self-care, and aggression, patients self-assessed worse than doctors’ assessments. On average, patients were assessed as doing better according to doctors’ assessments in both the conventional as well the newer medication group. From a patient point of view, there was a deterioration at visit V6 towards visit V0 in the conventional medication group for the activities and self-care sub-scores as well as for the total score. Regarding the pre-post differences in the sub-scores of the PSP questionnaire and the PSP self-rating, significant distinctions were found between the groups. Based upon doctors’ assessments of aggressive behaviour, there was a significant distinction in the pre-post difference between V4 and V0 in favour of conventional medication. Based upon self-assessments of socially useful activities, the distinction in the pre-post difference between V6 and V0 was significant in favour of the newer medication.

### Psychopathology, Quality of Life, Cognition, and the ICF

Doctors’ and patients’ assessments were positively correlated, that is, if doctors assessed patients as having a high value for PSP, the value assigned by patients was also high (Table 3). The activities and social relationships subscales as well as total scores correlated with the subscales of SF-36 and the total score. In cases where patients’ PSP conditions were assessed negatively by doctors, patients self-assessed their condition by SF-36 as not well and vice versa. The physical sub-scores of SF-36 and aggressive behaviour showed only occasionally significant correlations. Both scales were negatively correlated. If patients’ self-assessments required intensive support, the Area under the curve (AUC) value of their mental sum scale was low. Another chart showed AUC values of the mental sum scale compared with AUC values of the PSP total score. Both scores were positively correlated, that is, patients with high mental sum scales also showed high values in their self-rated PSP total score and vice versa. PSP and PANSS were significantly correlated in nearly all subscales. Specifically, the negative scale and aggressive behaviour were not correlated. The AUCs of the positive scale and the PSP sub-score activities were positively correlated, that is, if doctors assessed patients as having a high value for the positive scale, the value for activities was also high per doctors’ assumptions.

**Table 3:** Correlation (r) and Significance (s) between baseline values of PSP and PSP self rating

		PSP baseline			
	Activities	Relationships	Self-care	Aggression	Total score
Activities	r = 0.22591	r = 0.25101	r = 0.07168	r = −0.03276	r = −0.24804
	s = 0.0143	s = 0.0063	s = 0.4425	s = 0.0…	s = 0.0…
Relationships	r = 0.21096	r = 0.33122	r = 0.22253	r = −0,03790	r = −0.21721
	s = 0.0224	s = 0.0003	s = 0.0159	s = 0.0…	s = 0.0…
Self-care	r = 0.15643	r = 0.25589	r = 0.27222	r = −0.03818	r = −0.30325
	s = 0.0921	s = 0.0054	s = 0.0030	s = 0.0…	s = 0.0…
Aggression	r = 0.02060	r = 0.13825	r = 0.07977	r = 0.31700	r = −0.10092
	s = 0.8255	s = 0.1372	s = 0.3926	s = 0.0…	s = 0.0…
Total score	r = −0.15791	r = −0.14262	r = −0.02254	r = 0.00728	r = 0.23378
	s = 0.1618	s = 0.2069	s = 0.8427	s = 0.0…	s = 0.0…

The AUCs of the positive scale and the PSP total score were negatively correlated. If doctors assessed patients with a high value for the positive scale, the assessment for the total score was low; patients required intensive support. With a decreasing positive value, the total score was also lower. Doctors’ assessments of patients regarding PSP and PANSS showed a uniform picture.

All correlations between the sub-scores for the PSP and Mini-ICF-Rating (Table 4) were significant for baseline values as well as for AUC values. Here again, the correlations during treatment and post-treatment were high, as were the baseline values.

**Table 4:** Correlation between baseline values and AUC values of PSP furthermore between baseline values and AUC values of PSP selfrating and mini-ICF rating

PSP	Activities	Relationships	Self-care	Aggression	Total score
PSP baseline					
Mini-ICF rating baseline	0.50814	0.62658	0.45137	0.39203	−0.55988
	S < 0.0001	S < 0.0001	S < 0.0001	S < 0.0001	S < 0.0001
PSP AUC					
Mini-ICF rating AUC	0.60063	0.68296	0.60462	0.50711	−0.67629
	S < 0.0001	S < 0.0001	S < 0.0001	S < 0.0001	S < 0.0001
PSP self rating baseline					
Mini-ICF rating baseline	0.12986	0.30444	0.18136	0.09779	−0.19339
	S = 0.16850	S = 0.0010	S = 0.0535	S = 0.3006	S = 0.0920
PSP self rating AUC					
Mini-ICF rating AUC	0.22254	0.32602	0.22530	0.24599	−0.18063
	S = 0.01730	S = 0.0004	S = 0.0159	S = 0.0083	S = 0.1159

For the baseline values, doctors’ assessments for the Mini-ICF-Rating correlated significantly only for the relationship sub-score. The correlation between the Mini-ICF-Rating and sub-scores of the PSP self-rating during treatment and post-treatment were all significant but not as strong as the correlation to the PSP.

The baseline and AUC correlations between PSP, PSP self-rating, and cognitive parameters were not found. Most correlations were found in the area of PSP self-care. Thus, there was no relation between cognition and psychosocial functioning.

### Social Life, Sexual Functioning, and Well-Being Under Neuroleptics

Assisted living and PSP total scores, as well as PANSS total scores, were significantly correlated (i.e. patients with a lower total score for PSP and a higher total score for PANSS were more in need of assisted living (Table 5).

**Table 5:** (**a**) Correlation between PSP and assisted living. (**b**) Correlation of PSP and sociodemography

a.						
	Activities	Relationships	Self-care	Aggression		Total score
Assisted living	0,20533	0,03470	−0,07467	0,51310		−0,21486
b.						
Sociodemographic status	Occupation	Education	Relationship	Housing	Children	Assisted living
PSP total score	−0.00773	+0.02563	+0.19306	−0.13556	−0.16844	+0.20533*
PSP selfrating total score	−0.17577	−0.04736	+0.00885	−0.10031	−0.03302	−0.07467

Regarding baseline values, there are significant correlations between PSP self-care subscale and the subscales of the Derogatis scores (an Interview for sexual functioning from LR. Derogatis [6] on sexual cognition/fantasy and sexual experience, as well as the DISF-SR total score (Table 6). In addition, the correlation between the overall value of the PSP and the sexual drive/relationship subscale was also significant. The sub-scores of the DISF-SR were negatively correlated with the PSP self-care subscale. This means that if – from a doctor’s point of view – the patient needed more support in the area of self-care, the patient was less sexually active; conversely, if – from the doctor’s point of view – the patient had less difficulty in self-care, the patient was more sexually active. The overall value of the PSP questionnaire correlated positively with the subscale of sexual drive/relationship, but the statement regarding the relation with the scores remained the same. The less support a patient required the more sexually active the patient was in the area of sexual drive/relationship.

**Table 6:** Correlation between baseline values of PSP and DISF-SR (T-Scores)

	Activities	Relationships	Self-care	Aggression	Total score
Sexual cognition/fantasy	0,06625	0,10,922	−0,23457	0,00175	−0,11512
Sexual arousal	0,04814	0,16134	−0,13667	0,06845	0,04031
Sexual behavior/experience	−0,10995	−0,21029	−0,31711	−0,09086	0,13284
Orgasm	−0,05617	0,03001	−0,15202	0,06974	0,10279
Sexual drive/relationship	−0,06170	−0,08676	−0,11768	0,09222	0,25429
Total score	−0,01916	0,07955	−0,22119	0,05381	0,01877

Significant baseline correlations were also significant for AUC correlations. PSP self-care correlated significantly with two additional dimensions (sexual arousal and orgasm) from the Derogatis questionnaire. Additionally, there were significant correlations between sexual drive/relationship and activities and between sexual experience and personal and social relationships. All significant correlations added to the baseline consideration were negative. The relation between the scores remained the same.

The main areas of PSP self-rating correlated with other dimensions as the main areas of the PSP. Personal and social relationships correlated negatively and significantly with the areas orgasm and sexual desire/partnership for both baseline and AUC values. Additionally, the main area of aggression correlated with sexual cognition/fantasy for baseline and AUC values. However, both correlations were positive (i.e., if the patient was more aggressive, the patient is also more sexually active and vice versa). For the baseline correlations, sexual arousal correlated significantly and positively with main area activities. Regarding AUC correlations, sexual arousal also correlated significantly and positively with self-care, whereby the AUC correlation opposed the rating given by the doctor.

For baseline values, there was only a significant correlation between total scores for the SWN-K and relationships. A significant correlation between some sub-scores of the PSP and the SWN-K scores was found after medication administration (thus, for the AUCs). Exceptions were the sub-scores for aggression (PSP) and self-control (SWN-K).The sub-scores and the total scores of the SWN-K were negatively correlated with the sub-scores of the PSP and positively correlated with the total scores for the PSP. This means that patients who demonstrated a higher level of wellness had less difficulties, and vice versa.

Except for aggression, the sub-scores and total scores of the SWN-K were strongly correlated with the sub-scores and total scores of the PSP self-rating.

These correlations were more significant after medication administration.

## Discussion

This work provides a good random sample of many schizophrenic patients across Germany from several outpatient clinics. Included were patients from university centres as well as from non-university centres.

No discrepancies were found between the different treatment strategies regarding the administration of atypical or classical antipsychotics concerning psychosocial activities.

The main result of this study was that doctors assessed patients on the whole as well in the area of social activities, which was worse than patients self-reported. Furthermore, there were close relationships between PSP and quality of life, level of skills (ICF), and severity of disease (PANSS), but not in reference to cognitive changes, which is a new result in the PSP literature. An additional new finding indicated that a lowered psychosocial functioning was found to be correlated with a lower level of sexual activity and poor well-being under antipsychotics.

In a similar manner to our previous report [9], there were many differences between doctors’ and patients’ assessments regarding psychosocial functioning. For example, doctors assessed patients in total scores as well as in the area of activities worse than the patient self-reported. In the area of social relationships, self-care, and aggression, patients assessed themselves worse than doctors. According to doctors, patients felt better when treated with conventional medication as well as with atypical medication. From the patients’ point of view, this assessment was only shown for the treatment with atypical medication.

According to patients, there was a decline in well-being when treated with conventional medication in the areas of activities and self-care as well as in general. Regarding doctors’ assessment for aggressive behaviour, the difference was significant in favour of conventional medication. Regarding the self-assessment of socially useful activities, the difference was significant in favour of newer medication. In doctors’ point of view, aggressive behaviour improved with conventional medication; from the point of view of affected persons, socially useful activities improved with atypical antipsychotic medication.

When doctors assessed patients as having high psychosocial functioning, patients self-reported the same. Doctors’ assessment by means of the PSP conformed especially in the areas of social relationships and aggression to the self-assessment of the affected persons. Also, doctors’ assessment of psychosocial functioning by means of the PSP conformed to the self-assessment of the affected persons regarding their own quality of life by means of the SF-36. If the patients’ condition was assessed with the PSP as poor by doctors, patients assessed their condition with the SF-36 as the same. If patients assessed a higher supporting level for themselves with the PSP, psychological well-being assessed with the SF-36 was low and vice versa.

The PSP and PANSS were significantly correlated in nearly all subscales, which was comparable to previous findings [2, 5, 10]. Particularly, the negative scale and aggressive behaviour were not correlated. The results showed significant negative correlations between the PSP and PANSS in the negative, positive, general, disorganization, and arousal subscales, and less in emotional stress as well as delusion, social withdrawal, and hostility for PSP sub-scores. PSP self-assessment and the PANSS were correlated only in a few sub-scores and items. The negative scale and self-care were not correlated.

Psychopathology and psychosocial functioning were strongly related for patients with chronic schizophrenia [3]. PSP served as a reliable and valid instrument for the evaluation of psychosocial functioning for schizophrenic patients in acute stage of illness as well as during treatment. If doctors assessed patients with a high value for the positive scale in PANSS, the value for activities – as assessed by doctors using the PSP – was also high, but the assessed total score was low; therefore, patients in these situations required more intensive support. Assessments of patients by doctors with both the PSP and PANSS showed a uniform picture.

The relation between psychosocial functioning and the level of incapacity measured by the Mini-ICF-Rating was proven in all areas, especially as a result of doctors’ assessments and in its course of disease. With regard to both scales, the consistency of the PSP scale was confirmed. The relationship to ICF ratings today consists of a broad range of literature suggesting that the PSP as a short-term instrument can be easily used to assess complex psychosocial functioning [2, 7, 11].

Despite some reports contained in the small amount of literature concerning this topic [12], we found no relation between cognition and psychosocial functioning. An interesting question in this context would address which relations might be expected here. Previous reviews have already determined that cognitive functions affect employment, rehabilitation, work skills, and working behaviour in different ways. Symptoms are less important [13]. Another survey showed that cognitive training alone improves only neurocognitive abilities. Social skills, functional skills, and everyday behaviour improve only in combination with cognitive training and behavioural training, but not solely with cognitive training [14]. In a further survey by the same authors, it was stated again that cognitive training alone improves cognitive skills with less of an impact on psychosocial functioning. Functioning is improved by a combination of cognitive training and psychiatric rehabilitation [15].

Within the scope of the sociodemographic data, there was a relation between psychosocial functioning as well as psychopathology and assisted living. Patients prefer to be in assisted living if they experience low psychosocial functioning or intense symptoms. This fits well with the existing literature as well as reported clinical experiences. Thus, it can be debated whether or not the PSP is a good measure in reflection of rehabilitation and assistance needs for patients with schizophrenia. Since insurance companies will request more detailed reasons for such efforts in the future, the PSP could play an important role here.

A new finding reported recently in the literature explained that if patients – according to their doctors – had less difficulties in self-care, they were more sexually active (in regard to the subscale for sexual desire/relationship). If – in the doctors’ point of view – patients required more support with self-care, they were less sexually active. Furthermore, self-care correlated significantly with sexual arousal and orgasm. Additionally, there was a significant correlation between sexual desire/relationship and activities and between sexual experience and personal and social relationships.

For the self-assessment, personal and social relationships correlated negatively and significantly with orgasm and sexual desire/partnership. If patients assessed themselves as being in good relationships, their sexual interest declined. If patients assessed themselves as more aggressive, they consequently were more sexually active, and vice versa. If patients assessed themselves as active according to the PSP, their sexual arousal increased (significantly and positively). If patients self-assessed having good self-care, their sexual arousal increased (significantly and positively).

If patients had a higher level of well-being under antipsychotic treatment, their medically assessed psychosocial functioning was reported as much better, especially in regard to personal relationships; the opposite effect was also true. This also applied to self-assessment on functioning. Although the Subjective Well-Being Under Neuroleptics scale developed by Dieter Naber [16] was used in several studies (e.g., [17]) together with the PSOP, we did not find studies dealing with their relationship. Therefore, our result is of special interest.

Limitations of this study include the relatively small sample size and the significantly large drop-out rate. Discrepancies were also found in several answers regarding sociodemographic data, inter alia regarding school-leave qualifications, work, social status, and living. The relatively high drop-out rate, although somewhat higher than in previous effectiveness trials, resembles clinical reality insofar as a high proportion of patients admitted to psychiatric hospitals for treatment of an acute exacerbation of schizophrenia have to be switched to a second or third compound before an effective treatment is determined for each individual patient. These results certainly need confirmation in a larger patient cohort. However, further analysis of the factors that contribute the PSP is needed. These factors could be certain psychopathological features. Larger studies in different patient populations are needed [18, 19].

Together with the previous studies, the PSP scale is demonstrated as a reliable, valid, and well-practicable instrument for assessment of psychosocial abilities in this study. This paper shows a relationship between psychosocial abilities on the one hand and symptoms, medication, quality of life, incapacities, severity of disease, assisted living, sexuality, and well-being under medication on the other hand. This was mainly demonstrated only in doctors’ assessments versus self-assessment regarding psychosocial abilities. There was no correlation between psychosocial abilities and cognition. Some of this has already been shown in past studies. Differences have been detected in doctors’ judgement compared to those of the affected people. To scientifically learn about patients’ points of view regarding various topics is increasingly useful. The same applies for patients’ views on the conception of topics and questioning related to scientific works.
