# Correction to: Assessment of Psychosocial Functioning in a Large Cohort of Patients with Schizophrenia

**DOI:** 10.1007/s11126-022-09977-4

**Published:** 2022-03-12

**Authors:** C. Kossmann, J. Heller, M. Brüne, C. Schulz, M. Heinze, J. Cordes, B. Mühlbauer, E. Rüther, J. Timm, G. Gründer, G. Juckel

**Affiliations:** 1 Department of Psychiatry, Ruhr University Bochum, LWL University Hospital, Alexandrinenstr. 1-3, 44791 Bochum, Germany; 2 Department of Molecular Neuroimaging, Central Institute of Mental Health, Medical Faculty Mannheim, University of Heidelberg, Mannheim, Germany; 3 Brandenburg Medical School, University Clinic for Psychiatry and Psychotherapy, Immanuel Klinik Rüdersdorf, Rüdersdorf, Germany; 4 Department of Psychiatry and Psychotherapy, Medical Faculty, Heinrich-Heine-University, Düsseldorf, Germany; 5 Department of Pharmacology, Klinikum Bremen Mitte, Bremen, Germany; 6 Department of Psychiatry and Psychotherapy, University of Göttingen, Göttingen, Germany; 7 Competence Centre for Clinical Trials–Biometry, University of Bremen, Bremen, Germany

## Correction to: Psychiatric Quarterly (2021) 92:177–191 https://doi.org/10.1007/s11126-020-09773-y

The article “Assessment of Psychosocial Functioning in a Large Cohort of Patients with Schizophrenia”, written by C. Kossmann, J. Heller, M. Brüne, C. Schulz, M. Heinze, J. Cordes, B. Mühlbauer, E. Rüther, J. Timm, G. Gründer, G. Juckel, was originally published Online First without Open Access. After publication in volume 92, issue 1, page 177–191, the author decided to opt for Open Choice and to make the article an Open Access publication. Therefore, the copyright of the article has been changed to ©The Author(s) 2022 and the article is forthwith distributed under the terms of the Creative Commons Attribution 4.0 International License, which permits use, sharing, adaptation, distribution and reproduction in any medium or format, as long as you give appropriate credit to the original author(s) and the source, provide a link to the Creative Commons licence, and indicate if changes were made. The images or other third party material in this article are included in the article's Creative Commons licence, unless indicated otherwise in a credit line to the material. If material is not included in the article's Creative Commons licence and your intended use is not permitted by statutory regulation or exceeds the permitted use, you will need to obtain permission directly from the copyright holder. To view a copy of this licence, visit http://creativecommons.org/licenses/by/4.0/. Open Access funding enabled and organized by Projekt DEAL.

The original article has been corrected.

